# Epidemiology of Patient Harms in New Zealand: Protocol of a General Practice Records Review Study

**DOI:** 10.2196/resprot.6696

**Published:** 2017-01-24

**Authors:** Susan M Dovey, Sharon Leitch, Katharine A Wallis, Kyle S Eggleton, Wayne K Cunningham, Martyn I Williamson, Steven Lillis, Andrew W McMenamin, Murray W Tilyard, David M Reith, Ari Samaranayaka, Jason E Hall

**Affiliations:** ^1^ Department of General Practice and Rural Health Dunedin School of Medicine University of Otago Dunedin New Zealand; ^2^ Department of General Practice and Primary Health Care Faculty of Medical and Health Sciences University of Auckland Auckland New Zealand; ^3^ RCSI Medical University Bahrain Adliya Bahrain; ^4^ Te Ngae Medical Centre Rotorua New Zealand; ^5^ Department of Women's and Children's Health Dunedin School of Medicine University of Otago Dunedin New Zealand; ^6^ Department of Preventive and Social Medicine Dunedin School of Medicine University of Otago Dunedin New Zealand; ^7^ Best Practice Advocacy Centre Inc Dunedin New Zealand

**Keywords:** general practice, patient harm, safety, retrospective studies, electronic health records, New Zealand

## Abstract

**Background:**

Knowing where and why harm occurs in general practice will assist patients, doctors, and others in making informed decisions about the risks and benefits of treatment options. Research to date has been unable to verify the safety of primary health care and epidemiological research about patient harms in general practice is now a top priority for advancing health systems safety.

**Objective:**

We aim to study the incidence, distribution, severity, and preventability of the harms patients experience due to their health care, from the whole-of-health-system lens afforded by electronic general practice patient records.

**Methods:**

“Harm” is defined as disease, injury, disability, suffering, and death, arising from the health system. The study design is a stratified, 2-level cluster, retrospective records review study. Both general practices and patients will be randomly selected so that the study’s results will apply nationally, after weighting. Stratification by practice size and rurality will allow comparisons between 6 study groups (large, medium-sized, small; urban and rural practices). Records of equal numbers of patients from each study group will be included in the study because there may be systematic differences in patient harms in different types of practices. Eight general practitioner investigators will review 3 years of electronic general practice health records (consultation notes, prescriptions, investigations, referrals, and summaries of hospital care) from 9000 patients registered in 60 general practices. Double-blinded reviews will check the concordance of reviewers’ assessments. Study data will comprise demographic data of all 9000 patients and reviewers’ assessments of whether patients experienced harm arising from health care. Where patient harm is identified, their types, preventability, severity, and outcomes will be coded using the Medical Dictionary for Regulatory Activities (MedDRA) 18.0.

**Results:**

We have recruited practices and collected electronic records from 9078 patients. Reviews of these records are under way. The study is expected to be completed in August 2017.

**Conclusions:**

The design of this complex study is presented with discussion on data collection methods, sampling weights, power analysis, and statistical approach. This study will show the epidemiology of patient harms recorded in general practice records for all of New Zealand and will show whether this epidemiology differs by rural location and clinic size.

## Introduction

### Prior Related Research

Harming people who seek help from health services causes distress in both harmed patients and in the health care workers involved [1]. It is also morally wrong and costly, posing an often avoidable burden on already stretched health systems. Knowing where and why most harm occurs will enable patients to make informed decisions regarding the risks and benefits of treatment options and enable health care providers to improve systems and processes to deliver safer care.

Reviewing patient records as a methodology for understanding patient safety started in US hospitals more than 20 years ago and has been repeated in many countries, including New Zealand, but has extended beyond hospital care in only one country to date (the Netherlands [2]) [3-6]. Epidemiological research in general practice is important for advancing health systems safety [7] because the overwhelming majority of health care is delivered in primary care [8]. Health care delivered in general practice is known to harm patients, and many safety incidents identified in hospitals originate in primary care [9-12]. Conversely, many harms originating in hospitals may not become apparent until after discharge. The burden of patient harms on health systems is mainly from frequent repetitions of minor incidents rather than from rare extreme events [13].

The development of initiatives to protect patients from harm in general practice has been constrained by a lack of knowledge about the epidemiology of harm: the types of patient harms that occur, their likelihood of occurrence, severity, and degree of preventability. The only available general practice epidemiological patient safety study that has been conducted using the records review method found 211 incidents in 1 year of reviewed records of 1000 patients from 37 general practices [2]. No incidents resulted in severe harm or death, leading the authors to conclude that general practice is relatively safe. Although this is a critical early study, other research gives a different perspective.

In New Zealand, primary care medicine is typically provided by vocationally trained general practitioner (GP) doctors who care for patients of all ages and with all conditions. Although other medical specialists (including pediatricians and general physicians) occasionally work in general practice, they are most often employed in other settings. Our analyses of primary care treatment injury claims to the Accident Compensation Corporation in New Zealand [14] and of malpractice claims in the United States [15] show that health care delivered outside hospital settings can result in severe harm and death. Not all injured patients initiate malpractice suits or lodge claims for compensation for treatment injuries [16], so claims data underestimate both prevalence and incidence of these events. We therefore aim in this study to define the epidemiology of patient harms detectable in general practice records using retrospective record review methodology.

We recently completed a feasibility study to prepare for this proposed study. In the feasibility study, we derived information for calculating sample sizes for the full study and developed a workable rule-based definition of “harm.” We also discovered that the reviews needed to be completed by GPs as nonmedical reviewers were unable to adequately interpret general practice records and identify patient harm. We developed forms and processes to collect the study data.

### Hypotheses

As well as the need for descriptive epidemiology research, we aim to address unresolved questions relating to the influence of practice size and location on patient safety. In the last decade, reports of better quality care by higher-volume hospitals have fueled a shift of services from smaller to larger hospitals internationally [17] and mergers of small general practices into larger centers are now occurring in New Zealand [18]. If larger general practices were shown to be safer (or less safe) than smaller ones, this would have important implications for the future organization of general practice. Different types of harm might occur in different kinds of practices. For example, patients attending smaller practices may experience harm related to clinician availability, while patients of larger practices may be harmed by more complex communications processes. This study will therefore test the hypothesis that there is no difference between small, medium-sized, and large general practices in the epidemiology of patient harm detectable from general practice records.

The research will also test the hypothesis that there is no difference between rural and urban general practices in the epidemiology of patient harm detectable from general practice records. Patients of rural practices face the obvious disadvantage of greater distance from medical services than patients of urban general practices and distance from services is a recognized (but not measured) safety risk [19]. Rural patients may experience more harm than patients of urban practices related to limited access to hospital and specialty care, but no research has yet shown that rural patients experience more or different health care harms than urban patients [20]. In New Zealand, rural patients have reported concerns about primary health care costs, lack of access to doctors in emergencies, and inappropriate early discharge from hospitals [21].

We therefore aim in this study to provide the epidemiology of harms detectable from general practice records and to test whether there are epidemiological differences in recorded harms by practice size and location.

## Methods

### Study Design

**Figure 1:**
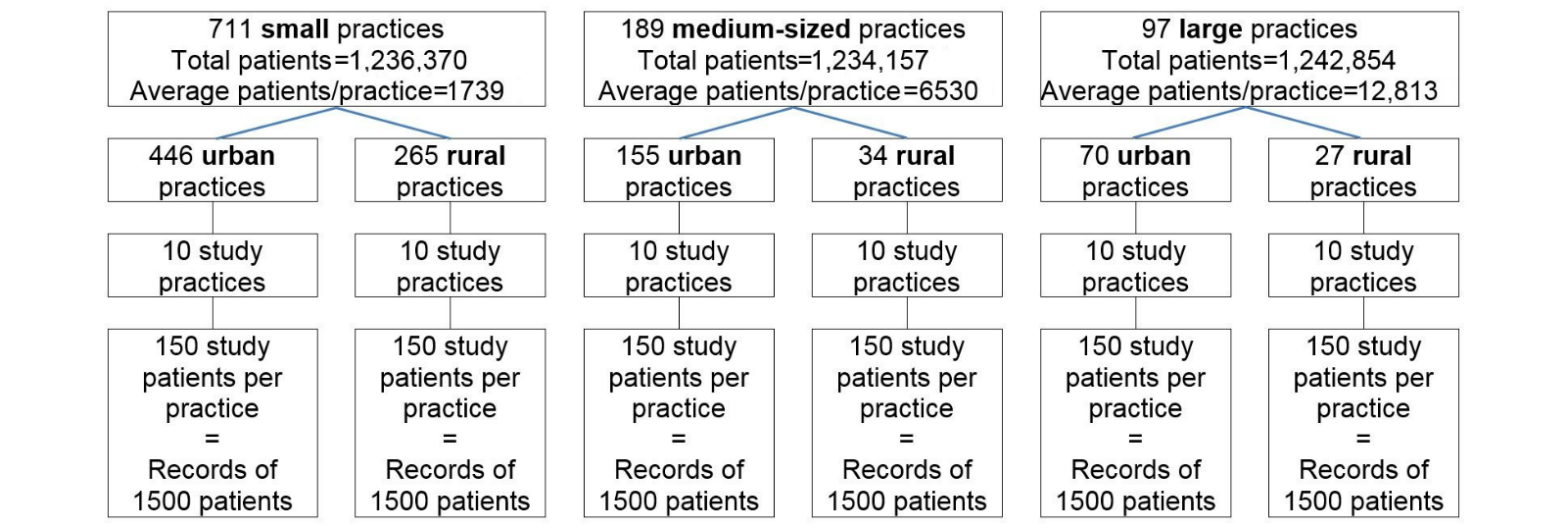
Study design using Primary Health Organization data from the third quarter, 2013.

The study design is a stratified, 2-level cluster, retrospective records review study (shown in Figure 1). New Zealand has a robust national database of enrolled general practice patients. Patients are incentivized to enroll with one general practice as unenrolled patients pay considerably more to receive general practice care. Patients are identified by their National Health Index (NHI) code, a unique alphanumeric identifier assigned to everyone using health services in New Zealand. General practices are required to advise their local Primary Health Organization quarterly of their enrolled patients using patients’ NHI codes. Practices receive a capitation payment based on this information and are penalized for providing incorrect information, so accuracy is paramount for both practices and primary health organizations.

### Study Population

We developed the study design using the enrollment database from the third quarter of 2013 because this was the most recent data available when the study was planned. Each quarter, earlier versions of the database are overwritten, so study general practices have now been randomly selected from practices in the fourth quarter 2014 Primary Health Organization database because this was the latest available data before the study started. The sample frame includes all New Zealand general practices, except the 2.85% of all general practices (29/1018) based solely in aged care residential facilities, universities or polytechnics, or specialty practices such as sports medicine, men’s health, or appearance medicine clinics. We excluded these clinics because they provide targeted primary care to specific populations and their inclusion could misrepresent typical general practice. We also excluded general practices that our data extraction software was unable to access.

From randomly selected general practices consenting to participate in the study, we will draw a random selection of patients enrolled at the midpoint of the study period, July 1, 2012. The number of patients whose records will be reviewed from each participating practice will vary according to the number of enrolled patients in the practice but will total 1500 for each of the 6 location and size study groups (for a total of 9000 randomly selected general practice patients to be studied). This will oversample minority groups compared with proportional selection, ensuring all group-specific estimates have reliable levels of precision.

### Study Period

The study period is the calendar years 2011, 2012, and 2013. Patients’ anonymized medical records for the 3 calendar years will be extracted. All randomly selected patients will be included for analysis even if they were not enrolled for the entire study period, to capture people who were born, changed practices, or died during the study period. The analysis will adjust for patient-years enrolled in study practices during the 3 study years.

### Power Calculation

From our feasibility study, we calculated that a randomly selected sample of 1345 patients in each study group will allow 5% differences in the proportion of patients harmed by their health care to be defined between urban and rural, and small, medium-sized, and large practices, with alpha=.05 and power=.80. Neither harm severity nor intraclass correlation was among the assumptions in our power calculations. We will include the records of all randomly selected patients, even if they had no recorded health care encounters during the study years.

### Recruitment

Although the power calculation is based on patient numbers, we wish to ensure that a nationally representative geographic distribution is achieved by the sampling process. We have therefore invited participation in the study of 12 randomly selected practices from each of the 6 study groups shown in Figure 1. We expect that 10 practices from each group will agree to participate, for a total study group of 60 general practices. This expectation is based on the assumption that most New Zealand general practices exclusively use electronic health records, with approximately 80% using the Medtech system [22]. Randomly selected practices will be excluded from the study if they do not use this records management system as we do not have funding to design data abstraction tools for the many different software packages used by the other 20% of general practices.

To encourage participation, the study has been endorsed by the Royal New Zealand College of General Practitioners as an audit activity, and participation counts toward the audit requirement for the maintenance of professional standards recertification program for participating GPs. No other incentives will be offered to participants.

Practices will receive the results from their practice as soon as their patient reviews are complete, and at the end of the study they will be advised of the final results.

### Definitions of Outcomes and Explanatory Variables

#### Patient Harm

**Figure 2:**
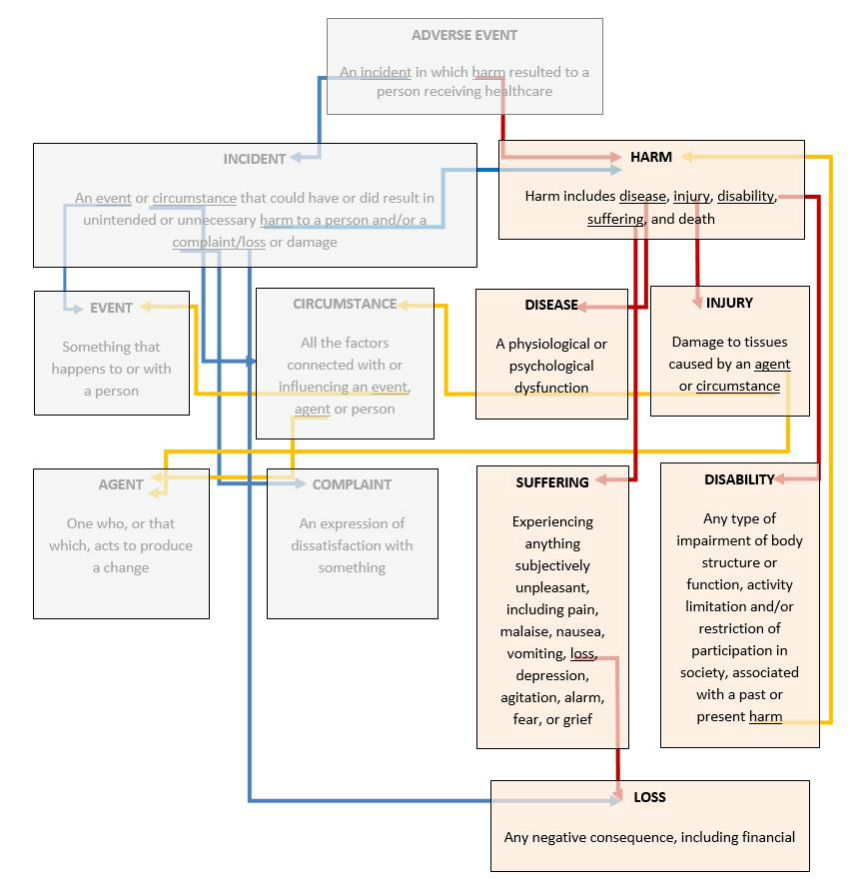
Relationship between patient safety terms. Red arrows indicate relationships between key terms addressed in this study. Blue arrows and opaque boxes indicate definitions and relationships between terms that are not the subject of this study. Yellow arrows indicate where terms are used to describe both “incidents” and “harm.”

Patient harm is defined as physical, emotional, or financial negative consequences to patients directly arising from health care, beyond the usual consequences of care and not attributable to patients’ health condition. Figure 2 shows our operational definition of patient harm, derived from work undertaken by the Australian Council for Safety and Quality in Health Care [23].

#### Harm Preventability

Harm preventability assessment requires GP insight. While some harms are clearly preventable (eg, a patient with a history of specific adverse drug reaction experiencing another adverse reaction after being prescribed the same medicine) or not preventable (eg, a patient having an adverse reaction to an appropriately prescribed drug, without a previous history of such reactions), there is a considerable gray area in general practice, where risks and benefits of treatments have to be balanced and sometimes harm is knowingly risked in the interests of avoiding potentially greater or more permanent harm [24]. We will use the standard of a “reasonable” doctor to focus reviewers’ consideration of preventability, as in hospital records review studies [3,9] and our feasibility study. Preventability will be coded using the definitions developed by McKay et al [25] as “not preventable and originated in secondary care,” “preventable and originated in secondary care OR not preventable and originated in primary care,” “potentially preventable and originated in primary care,” “preventable and originated in primary care,” or “not preventable, standard treatment.”

#### Harm Severity

Harm severity will also be reviewed by the GP investigators, who will make subjective assessments of severity from “minor” to “severe” (Table 1). We will collect data specifically on whether harms resulted in death, hospital admission, emergency department contacts, additional general practice visits, or additional treatments.

**Table 1:** Harm severity assessment with examples.

Harm severity	Examples
Minor	Minor drug adverse effects (eg, nausea, rash), grazes, bruises and lacerations, and inconvenience to patients caused by processes of care, such as being given the wrong prescription.
Moderate	Ongoing morbidity attributable to omissions in care management (eg, ongoing poor diabetes control, untreated anemia, repeated abortions) and fracture of minor bones (eg, ribs).
Severe	Severe harms include renal failure, pulmonary embolism, myocardial infarction, peptic ulcer perforation, delayed cancer diagnosis, morphine overdose, and fracture of long bones.
Death	Death

#### Practice Size

Practice size is defined by the number of registered patients, rather than the number of clinicians, an approach used in some other studies [26,27]. Small, medium-sized, and large general practices are defined in Figure 1 by tertiles of total number of enrolled patients in all New Zealand practices. There will be some heterogeneity between practices within each size group. In theory, random selection of practices will even out the effects of this heterogeneity.

We considered the effect of the few New Zealand general practices that do not employ any GPs at the time of selection. The number of these practices is unknown. We assume that all such practices will fall into the “small” size group and the random selection process will allow their entry into the study sample. We will collect descriptive data from study practices, including staff composition, to allow analysis of differences between practice types in terms of harm probability or severity.

#### Rurality

Rurality is defined in New Zealand by the Rural Ranking Score developed in 1995 as an objective measure for allocating public funding to support the recruitment and retention of rural GPs and to assist the provision of after-hours care in rural and remote communities. By this measure, practices with a score of >35 capture the features of rurality that make them substantially different from urban general practices. Therefore, we will collect data about study practices’ Rural Ranking Score, if they have one, and we will also define rural and urban practices by their addresses in locations meeting the Statistics New Zealand definitions of urban and rural [28], with one exception. Practices in “independent urban communities” will be included in the *rural* general practice group as independent urban communities are smaller centers without many of the specialty services provided by large hospitals [29]. Many of the patients of general practices in these towns live in surrounding rural areas.

### Collection of Study Patient Records

**Figure 3:**
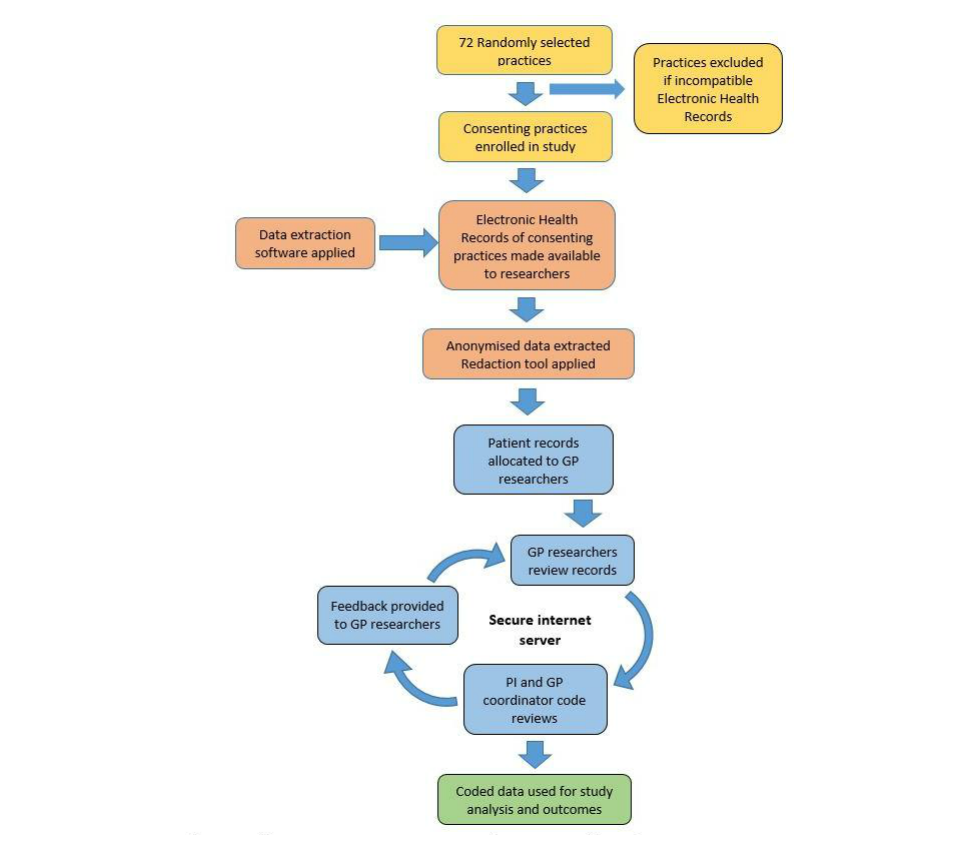
Flowchart of study processes. Color key: yellow=practice engagement, orange=data extraction, blue=data review, and green=analysis. GP: general practitioner; PI: principal investigator.

Figure 3 shows the study’s data processes. Study patients’ records will be extracted electronically from the computer systems of participating general practices. Extracted patient records will include the dates for, and notes about every contact with the practice, all prescriptions and investigation results, and discharge summaries from hospitals. We may be unable to review referral letters and some other important information that is stored in portable document format (“.pdf”). Extracted data will be reviewed and used to complete the study data form. The anonymized extracted records, along with reviewers’ assessments of harm, will be sent to the Dunedin School of Medicine, Department of General Practice and Rural Health.

### Data and Missing Data

Descriptive data will be collected from the general study practices at enrollment, including practice location (using both the Rural Ranking Scale and Statistics New Zealand definitions), size, services available, clinical staff composition, and clinical hours worked. This information will be held separately from the patient records and will not be visible to the GP reviewers.

Specially programmed software will interrogate the electronic health records of study practices, make a random selection of patients, and extract 3 years of data from these patients’ records. Extracted patient records will be allocated to reviewers, who will access them via a secure website. Security of personal information is recognized by strict privacy legislation in New Zealand [30], so no identifiable patient information will be collected. Patients will be allocated a numeric study identifier. Deidentifying redaction software will be used to strip names and addresses from records, as far as possible. Dates of birth will not be collected but patients’ ages (in years) during each study year will be recorded. Each record will have a data form attached, which will include study identifiers for the patient and practice, patients’ age, sex, ethnicity, and social deprivation using the geographically based NZDep Index. Free text will be used to record each harm that patients experience during the 3 study years, with drop-down boxes to record the preventability and severity of each harm. Multiple harms may be recorded for each patient. Where no harm details are entered by GP reviewers, patients will be recorded as having “no harm.”

As all study data are drawn from general practice records, their completeness will depend on the processes used in each practice. Our experience is that age, sex, event dates, prescriptions, and investigation results are almost always 100% complete as these data are ensured by the software. Ethnicity is variably recorded: we expect this to be missing from about 40% of records. The content of free text medical records depends entirely on the idiosyncratic practices of doctors and nurses: sometimes very detailed descriptions of patients’ journeys through health care are provided, but sometimes descriptions are sketchy.

As the goal of the study is to determine from general practice records the epidemiology of harm in a way that can be generalized to all New Zealand general practices, we plan to unconditionally accept for review all randomly selected records from participating practices. The only absolute requirement is that they are electronic records on software compatible with our extraction tool.

### Data Coding and Checking

We found from the feasibility study that harms were seldom explicitly stated in the records but were recognizable to GPs who were able to interpret patients’ journeys through the health care system after reading their general practice records.

All patient records reviews will be conducted by GPs who are currently clinically active. There are 8 GP reviewers distributed throughout New Zealand, including 5 who reviewed records in the feasibility study. Reviewer training includes an 8-hour interactive workshop before data collection, annual face-to-face meetings, reviews are critiqued and discussed in an online forum, monthly individual feedback is provided to each reviewer, and all reviewers have the opportunity to seek clarification about their work with the pharmacologist and study coordinators.

Most records in the proposed study will be reviewed by only 1 GP investigator, but a 5% random sample of records will have blinded review by a second GP to check concordance and identify areas where additional clarification and training may be needed (450 patient records). This was considered a sufficient number for reviewers to learn where discordant interpretations might happen and to develop consensus for frequently encountered harms. A final decision will be made by consensus. The blinded reviews will occur early in the study to ensure consistency between reviewers. GP investigators will be randomly allocated patient records to complete from any study practice, excluding practices from localities where the GP investigator has recently or currently worked.

Reviewers’ free-text notes describing patient harms will be coded by the principal investigator (SMD) and the GP coordinator (SL) using the international clinically validated Medical Dictionary for Regulatory Activities (MedDRA) 18.0. This dictionary was chosen for its scope of classifications. SMD and SL will code all harms together, working collaboratively to obtain consensus.

Because most harms in the feasibility study were related to medications, and in order to assist a sensitivity analysis, a pharmacologist will rereview a randomly selected 5% of study records searching specifically for medicine-related harms that may have been missed by the GP investigators owing to their lack of specific expertise in pharmaceuticals use and toxicology.

### Statistical Analysis

Survey data statistical analysis tools appropriate for the sampling design will be used by the study’s biostatistician (AS) to analyze abstracted data. These are the “svy” group of tools in Stata. Sampling weights will be used to accommodate the study design features that allow, for example, a higher probability of selecting a larger practice into the sample but a lower probability of selecting a patient from large practices for records review. Probabilities of harm, harm types, harm severity, and harm preventability will be calculated overall and for each practice group. Rates will be calculated using as denominators all patients, consultations, medications, and other health care activities (such as surgery) relevant to each harm-related activity. The analysis will describe and adjust for patient characteristics and harm type. Other data (such as event dates) will be used to interpret harms in light of external events, to help reviewers understand the chronology of events, and to estimate probabilities of specific types of harm.

We will use a mixed model analysis (the “xtmixed” group of tools in Stata) to explore hypotheses relating to harm differences associated with rurality and size. Mixed effects modeling is also necessary to accommodate multiple harms (with multiple scores for preventability and severity) for some patients.

### Ethics

Consent will be obtained from the general practices, not from individual patients. This research has been approved by the University of Otago human ethics committee (HD14/32). The Ngāi Tahu Research Consultation Committee has endorsed this research. As well as obtaining local ethics committee approval, we are seeking to have the researchers and participating practices protected by law, in the unlikely event our research reveals grievous malpractice. By the process set out in the Health Practitioners Competence Assurance Act 2003 [31], the Minister of Health will endorse the research as a protected Quality Assurance Activity, which protects the confidentiality of information and gives immunity from civil liability to people who carry out this research.

## Results

Funding for the study has been obtained from the Health Research Council of New Zealand. We have enrolled 46 eligible general practices into the study and downloaded from these practices the electronic health records of 9078 patients. Records review is currently under way and the first results are expected to be submitted for publication in late 2017.

## Discussion

### Aspects of the Study

This study will address the lack of epidemiological knowledge of patient harms in general practice by a comprehensive analysis of general practice electronic health records. The random sampling of first practices and then patients within those practices means the results will be generalizable across New Zealand. Exclusion from the study of 3% of ineligible clinics and 20% of clinics with inaccessible patient data may bias the results. We will consider potential biases in interpretation of study results. The scope of the sample is broad enough to address concerns regarding selection bias, in terms of both patient numbers (9000) and study duration (3 years). Using clinically active GPs to review clinical records is a strength of the study. Concordance will be measured by double-blinded reviews. Coding will be reliable, as it will be done collaboratively by a clinically active GP who also has a reviewing role (SL) and a general practice researcher (SMD).

This study addresses an important gap in patient safety research, which to date has largely ignored harm occurring in general practice. It is powered to determine whether harms to patients systematically differ by rurality or by practice size, because this information will assist clinicians, organized general practice groups, and funding agencies to understand and ameliorate the specific safety risks of different types of general practice.

### Perspectives

Information from the study will be of practical use to a wide variety of stakeholders. Patients will have new knowledge to inform decisions about treatment and type of practice to register in (with respect to size and location). The research will directly affect clinical decisions in general practices by alerting clinicians to the types of common and preventable patient harm likely in their specific type of practice (small, medium-sized, or large, and urban or rural). In addition, it will provide information at policy and organization levels. For example, it will inform public health safety agencies about the medicines commonly causing harm in the community (influencing decisions on their educational foci) and it will provide information to government and primary care groups about the relative safety of large and small practices, which may influence policy decisions regarding practice mergers.

### Practical Applications From Study Results

Based in general practice, but including harm from other settings referenced in general practice records, this research will provide an epidemiological base to a general practice patient safety trigger tool that will help health care become safer for patients. Triggers are circumstances associated with a higher likelihood of preventable harm than other health care situations. Trigger tools are used as a focused way of measuring care safety improvements for particular groups of patients but can also be used as prompts to additional caution in patients with certain characteristics, which this study will identify. To be effective, triggers must be sensitive (capture most harm situations happening in general practices) and specific (so that GPs do not to have to spend time investigating situations seldom associated with harm). Widespread trigger tool use is the most likely way for GPs to identify remediable safety threats in their practices in the medium term and into the future, but in our feasibility study we found that only 20% of the harms identified from a random sample of records would have been identified by the NHS Trigger Tool: 80% of harms would not. We found that many existing triggers were neither sensitive nor specific and therefore of little practical use to New Zealand GPs.

The study’s results will inform the development of triggers targeted to the most common and severe harmful situations patients experience in general practice. Although trigger tools are already recommended and used in several countries [32-34], they have typically not been grounded in epidemiological research. Instead, they have been based on opinions of high-risk areas of practice [32], so they may not be targeting the most problematic areas of practice. This is becoming apparent in hospital-based research [35] and is reflected in the results of our feasibility study. Protecting patients from health care–associated harm is important, and we intend for this study to advance that objective in general practice.

### Conclusions

Harm may be a good signal of overall quality of primary care, but further research is needed before this can be stated with confidence. In most general practices, there is no capacity for the managerial oversight or complex investigations that feature in hospital-based systems to protect patient safety. To date, general practice in New Zealand has largely been excused from engagement in the patient safety agenda because of beliefs that the frequency of serious harms in general practice is low. The proposed research will objectively test this belief, find out what harms primary care patients experience, and use the results to develop strategies for reducing serious and common harms to patients. This is the first step to addressing patient safety in the setting where most people receive most of their health care and, perhaps, improve quality.

## Abbreviations

GP: general practitioner
MedDRA: Medical Dictionary for Regulatory Activities
NHI: National Health Index

## Appendix

Multimedia Appendix 1 Funding peer review report: Health Research Council of New Zealand.
