# Supplementary material for: Epidemiology of Patient Harms in New Zealand: Protocol of a General Practice Records Review Study
Source: JMIR Res Protoc. 2017 Jan 24;6(1):e10. doi: 10.2196/resprot.6696 (PMC5296619; doi:10.2196/resprot.6696)
Supplement: Multimedia Appendix 1 [file resprot_v6i1e10_app1.pdf]

Health Research Council of New Zealand

Referee Report - Applicant version

Assessing Committee: .2014 HDAC Full HRC Ref# 14/185

Referee#: 62

PROPOSAL DETAILS:
14/185 Professor Susan Dovey
Patient Harms in New Zealand General Practices: Records Review Study
(University of Otago)

HEALTH SIGNIFICANCE	Score: B

I am not overly convinced of the significance. First, a Dutch study found no severe harm among 1000 patients. Whilst it is difficult to calculate a confidence interval about a zero proportion, the frequency is obviously low.
Second, as a reader of this application, I do not get a feel for what constitutes a harm. Nor by mild, moderate, serious. What would? Some examples might help the assessing committee. Further, some quantification of the impact on patients from mild, moderate and sever harms would be useful. Are we talking about half an hour of mild symptoms, a day in bed, a week off work, or permanent disability? Is there any comparator that could be used? (E.g. changes in EQ5D, SF36 or other scores – and how these compare to (say) an episode of gout, mild anxiety, etc. Or using disability weights from the recent GBD 2010 (Salomon et al, Lancet, 2012).) In a world where research has to be prioritised, and there are big health losses from NCDs, I am not confident that this research is addressing a key public health problem.
Third, the researchers state that ACC provides some data – but it is an underestimate. But could we at least have those results/prevalences for the assessing committee to peruse, and with the researchers view on how much of an undercount they might be. (I note also that some assumptions must have gone into the power calculations about prevalence. What were they? Were they for moderate, mild or severe?)
If the magnitude of health harm here is not that large, then there are still three (at least) redeeming features of this application in light of significance: 1) the research is ‘cheap’ compared to the standard $1.2 million HRC grant, so value for money is commensurately improved; 2) harm may be a good signal of overall quality of primary care (yes?); 3) New Zealand still has a role in generating evidence that is internationally useful – Dovey has a assembled an impressive team, and maybe this is something the HRC should invest in as a contribution to international knowledge as much as national knowledge.

SCIENTIFIC MERIT	Score: B

Noting above, generally good. But some examples of mild, moderate and sever harm would be useful.

DESIGN AND METHODS	Score: B

In many aspects very good, thorough and well thought through.

My two major quibbles (in addition to power to detect what?) would be:

- I am not convinced that just because 2 GPs in the pilot gave the same answer that this would apply to a

wider group of researchers.

- the 5% cross-checking of assessment (i.e. double scoring) sounds good. But how is it going to be used? If early in the research, it could be to increase research training and concordance. But if not, to what purpose? Will the study have enough power with (say) a 5% subsample as a sensitivity test for misclassification of the harm variable? How can this validation sample be usefully harnessed for study internal validity?

EXPERTISE AND TRACK RECORD OF THE RESEARCH TEAM	Score: A

Impressive.

OVERALL/GENERAL COMMENTS (To Applicant, HRC and Committee)	Overall Score: B

The assessing committee needs to be convinced of the 'burden' from GP harm. If the assessing committee is convinced of the significance of this harm, then the research is generally of a sound and high quality.

Printed at:..2014-02-24 21:57:11 - ref 10440

Health Research Council of New Zealand

Referee Report - Applicant version

Assessing Committee: .2014 HDAC Full   HRC Ref# 14/185

Referee#: 94

PROPOSAL DETAILS:

14/185   Professor Susan Dovey

Patient Harms in New Zealand General Practices: Records Review Study

(University of Otago)

HEALTH SIGNIFICANCE	Score: B

Study focuses on the quality. safety and accessibility of services criterion.

SCIENTIFIC MERIT	Score: C

I think the scientific merit is satisfactory but a number of issues related to the design suggest knowledge advancement will be somewhat limited.

DESIGN AND METHODS	Score: C

The principal reference used in the rationale for the study identifies 'errors of omission' as the greatest concern in relation to harm in ambulatory services. The proposal does not address this adequately and does not provide adequate information about how it will be measured and coded. Given this has been identified as a major concern it would have been expected that this would be addressed in the proposal. The validity and reliability of the coding process also needs to be addressed because the proposal implies a reasonably high level of subjective interpretation.

EXPERTISE AND TRACK RECORD OF THE RESEARCH TEAM	Score: C

The PI has led a feasibility study but has limited experience in leading a large study. The other investigators have fairly limited experience in conducting large studies.

OVERALL/GENERAL COMMENTS (To Applicant, HRC and Committee)	Overall Score: C

This study may be useful for identifying patient harm in GP settings but the proposal itself may have let the study down.

Printed at:..2014-01-06 12:19:58 - ref 10440

Health Research Council of New Zealand

Referee Report - Applicant version

Assessing Committee: .2014 HDAC Full HRC Ref# 14/185

Referee#: 39

PROPOSAL DETAILS:
14/185 Professor Susan Dovey
Patient Harms in New Zealand General Practices: Records Review Study
(University of Otago)

HEALTH SIGNIFICANCE	Score: A

The issue of harm to patients is important and little is known about it in the primary care setting.The potential contribution of increased knowledge about harm to health is substantial particularly if the preventability of harm can be understood and some hints at mechanisms that could be addressed in future evaluations and trials.

SCIENTIFIC MERIT	Score: A

What is particularly novel in this application is the attempt to establish associations between primary care structure and function and harm. This is critical to an understanding of the potential impact of the changes that are occurring in primary care on outcomes. The New Zealand primary care setting is unique and data from other countries cannot be applied so this study will advance knowledge with very high relevance to New Zealand and contribute substantially to the sparse literature on harm in primary care.
the aims are somwhat global and could be more clearly stated

DESIGN AND METHODS	Score: B

The design and methods is in general of a high standard. It would be important to better understand the results of the pilot study – could these be included in the rebuttal.
Coding of ethnicity is reported here to be poor but that is not my understanding from work in the north island. Could the authors reference the source for the statement about inaccuracy of ethnicity coding and comments on whether this is likely in all sites of the study (what are the sites exactly)? The role of ethnicity in harm is pretty important and it would be essential to ensure that enough M?ori and pacific peoples are included to generate meaningful results for these subgroups.
Some quality related questions arise on reading. Will there be an attempt to establish interrated reliability of the judgement of harm severity? (P 9 3rd para
Will informed consent be obtained from participants whose notes are reviewed?

EXPERTISE AND TRACK RECORD OF THE RESEARCH TEAM	Score: B

the PI is internationally recognised ofr work in avoiding harm research and part of an interantional collaboration about this topic. She is supported by a good mix of clinical and academic partners. If this proposal is funded i would expect it to be completed.

OVERALL/GENERAL COMMENTS (To Applicant, HRC and Committee)	Overall Score: A

this is an essential proposal for funding.

Printed at:..2014-02-25 10:10:47 - ref 10440

Health Research Council of New Zealand

Referee Report - Applicant version

Assessing Committee: .2014 HDAC Full HRC Ref# 14/185

Referee#: 18

PROPOSAL DETAILS:
14/185 Professor Susan Dovey
Patient Harms in New Zealand General Practices: Records Review Study
(University of Otago)

HEALTH SIGNIFICANCE	Score: B

The extent and nature of patient harms is unknown in primary care and this study will provide the epidemiology.
It will also provide information to develop a Trigger Tool suitable for use in NZ General Practice, which will be useful to measure harms over time.

SCIENTIFIC MERIT	Score: B

This is important research and is original in primary care

DESIGN AND METHODS	Score: B

I have some concern about the reproducibility of coding by the different GP reviewers. They state that 5% of charts will be randomly selected to be coded by a second GP - I would suggest that this may need to be weighted more to the initial months of the study or increased. Also it is unclear what training is proposed for the GP reviewers. Are the researchers going to set up a forum for GP reviewers to discuss difficult cases. This is what was done for the Adverse Drug Event Trigger Tool and proved invaluable, not only for sorting out discrepancies, but for writing down common definitions.
It would also have bee nice to see the data collection audit tool from the feasibility study.
There was not a good definition of what a trigger tool was and its use. It is a modified chart review using triggers to identify charts that should be examined more closely for detection of possible patient harms. It is not designed to detect all patient harms, but it requires fewer resources than full chart review. Its benefit lies in a systematic approach that can be used over time in a practice to measure patient harms. (see 1. Mary E Seddon, Aaron Jackson, Chris Cameron, Mary L Young, Linda Escott, Ashika Maharaj, Nigel Miller. The Adverse Drug Event Collaborative: a joint venture to measure medication-related patient harm. New Zealand Medical Journal. 25 January 2013, Vol 126 No 1368; ISSN 1175 8716 Page 9 URL: http://journal.nzma.org.nz/journal/126-1368/xxxx/)

The issue of preventability is also a vexed one - the original IHI trigger tool did not support classification based on preventability - their rationale was that definitions of what is preventable vary over time (decreasing the utility of the TT to record harms over time)- for example, Central Line Associated Bacteraemia was not thought to be preventable, until Pronovost and others showed that a rate of zero was attainable in 103 ICU in the states.
Are you planning on having any patients on your research group? The TT was designed to record harm from the patient's perspective, and some of the harms considered minor by doctors (e.g. prolonged nausea)

are important to patients.

EXPERTISE AND TRACK RECORD OF THE RESEARCH TEAM	Score: A

This is a team with experience and the track record to complete this study

OVERALL/GENERAL COMMENTS (To Applicant, HRC and Committee)	Overall Score: B

The timeline for completion - for publication of findings (3 years)seems generous, but each GP reviewer has quite a load 54/month.

Printed at:..2014-02-07 14:46:21 - ref 10440

DO NOT DELETE OR MODIFY THIS TABLE!			
**Applicant**	Professor Susan Dovey	**Respond by:**	---
**Assessing Committee**	2014 HDAC Full	**HRC Reference**	14/185
**Title of Research**	Patient Harms in New Zealand General Practices: Records Review Study		

We are grateful for the thoughtful reviews of the four referees. The referees acknowledge the usefulness and originality of the study's exploration of differences between practices of different size and urban/rural location, the importance of its design and the research team for translation of its findings, and the contribution this research can make to both New Zealand and international audiences. They also raised some important issues, addressed below.

Referee 62 was the only reviewer not convinced of the significance of the study, given the failure of the Dutch study to identify serious harms. The Dutch study's findings made no sense to us because serious harms feature in most primary care patient safety research. It is precisely *because* of that study's results that we designed our study with greater care, especially regarding sample size. The Dutch study had no power calculations and only 1000 patients.

To clarify the context for the proposed study, in our previous study of 6,007 ACC claims[1] 83% of primary care treatment injuries were assessed as minor, 12% were major, 4% were serious and 1% sentinel. We think the ACC treatment claims database may capture less than 1% of all treatment harms happening in New Zealand general practice, based on our feasibility study finding of 29% of randomly selected patient records showing evidence of harm. We therefore have no confidence that the distribution of harms represented in the ACC database reflects the epidemiology of harms in general practice, generally. However, **there is sufficient accumulated evidence to suggest that harm arising from primary healthcare is a significant public health issue that needs to be better understood through research**.

Referees 39 and 62 requested more information about the feasibility study data. In the feasibility study, 8% of harms were assessed by the study GPs as severe, 37% as moderate and 55% as minor. Severe harms included death, renal failure (3 cases), pulmonary embolism, myocardial infarction, peptic ulcer perforation, delayed cancer diagnosis, morphine overdose – all happening as a consequence of healthcare provided or omitted (following the definition of 'harm' we arrived at through qualitative study). Fractures of major bones (e.g. femurs) were considered severe and of minor bones (e.g. ribs) moderate. Ongoing morbidity attributable to omissions in care management (e.g. ongoing poor diabetes control, untreated anaemia, repeated abortions) was considered moderately severe harm. Minor harms included rashes, minor drug adverse effects, grazes, bruises and lacerations, and inconvenience to patients caused by processes of care, such as being given the wrong prescription. Harm *severity* was not among the assumptions made in our power calculations: they were constructed using the feasibility study's estimates of harm prevalence and general practice size and location.

We agree that harm *"…may be a good signal of overall quality of primary care"* (Referee 62) but further research is needed before this can be stated with confidence. Certainly we have found that harm is more readily identifiable when more information is contained in patients' records: more identifiable harm may signal higher quality care. **Harm currently is almost always not specifically identified** but can be inferred from the longitudinal record. In most general practices there is no capacity for the managerial oversight or complex investigations that feature in hospital-based systems to protect patient safety. To date, general practice in New Zealand has largely been excused from engagement in the patient safety agenda, because of beliefs that *"…the frequency* [of serious harms] *is obviously low"* (Referee 62). The proposed research offers the opportunity to objectively test this belief, find out what harms primary care patients experience, and use the results to develop strategies for reducing serious and common harms to patients. This is the first step to addressing patient safety in the setting where most New Zealanders receive most of their health care and, perhaps, improve quality.

We will not be seeking individual consent from patients whose records will be reviewed for the study (Referee 39). We have completed many studies of general practice records where requirements for individual patient consent were waived by the New Zealand Health and Disability Ethics Committee because, as in the proposed study, individual patient data were completely de-

DO NOT DELETE OR MODIFY THIS TABLE!			
**Applicant**	Professor Susan Dovey	**Respond by:**	---
**Assessing Committee**	2014 HDAC Full	**HRC Reference**	14/185
**Title of Research**	Patient Harms in New Zealand General Practices: Records Review Study		

identified. In this study, the data abstraction program will assign a unique patient/practice number that relates to no other existing identifier. Patients are not only on the research team (Referee 18), the proposed study is **patient led**. The PI (Dovey) is an informed patient, with the authority to challenge the medical doctors regarding their assumptions of patients' perspectives.

We agree with Referee 39 that *"The role of ethnicity in harm is pretty important…"*. Ethnicity is also a complex construct that has been vexed for general practice researchers for many years[2] such that we mistrust the often conflicting ethnicity data in New Zealand's various health databases and records sets. Although we expect that random sampling of general practices nationally will deliver representative samples of patients of different ethnic groups, this is not guaranteed. We will collect available ethnicity data and conduct a sub-analysis by ethnicity but our power studies did not take ethnicity into account and resource constraints prohibit oversampling from Māori and Pacific peoples to guarantee meaningful subgroup analysis. As we state in the application, the interests of different ethnic groups may best be served by conducting separate investigations, using this preliminary study as a starting point.

Referees 18 and 62 expressed concerns about the training and reproducibility of coding by different GP reviewers. As suggested, the 5% double-checking will be a component of the meetings and training sessions and will be concentrated in the first 6-9 months of data collection. We will use the kappa statistic to test inter-rater reliability (Referee 39). Of the 8 clinician investigators in this study, 6 were involved in the feasibility study, reviewing general practice records and developing the definitions and processes for the proposed research. Through this process and training under the feasibility study grant we worked out definitions of harm and developed agreement about how to measure severity and preventability. In the proposed study we have budgeted for meetings (which will include training sessions) that all 8 clinician investigators will attend. As the investigators are located throughout New Zealand they will also have frequent contact with the Dunedin-based researchers via email and telephone calls. We have already built a community of researchers in the feasibility study. That community will be extended to include Eggleton and McMenamin in the proposed project, so the *"…wider group of researchers"* Referee 62 refers to is not much wider.

The GP reviewers will report the types of patient harm they observe in the records in free text but **they will not be responsible for coding** (Referee 94). The Dunedin-based researchers who will have easy ongoing access to each other to check coding consistency, will code and classify these events using a taxonomy developed for primary care patient safety research internationally.[2,3] We appreciate that the desirability of preventability coding is debated (Referee 18), but we think it is a useful consideration and we have found that we can do it with reasonable reliability. Mostly in general practice records preventability relates to whether, given available knowledge, a harm should have been averted.

Reviewer 18 rightly comments that trigger tools are not intended to detect all patient harms but other **global** primary care trigger tools were intended to detect the most important harms in general practice and **they were developed without knowing the epidemiology of harm**, and therefore what the most risky situations actually are and what triggers should be used. Our study will provide a robust evidence base for a New Zealand global primary care trigger tool.

1. Wallis K, Dovey S. No-fault compensation for treatment injury in New Zealand: Identifying threats to patient safety in primary care. *BMJ:Qual Safety* 2011; 20: 587-91.
2. Kljakovic M. Is it easy collecting ethnicity data in general practice? *NZ Med J* 1993; 106: 103-4.
3. Dovey SM, Meyers DS, Phillips RL, Green LA, Fryer GE, et al. A Preliminary Taxonomy of Medical Errors in Family Practice. *Qual Safety Health Care* 2002; 11: 233-8.
4. Klemp K, Dovey S, Valderas J, Godycki-Cwirko M, Rohe J, et al. The development of a patient safety incident classification for primary care on the basis of existing systems. *Eur J Gen Pract* [In Press]
